# An Optogenetic Kindling Model of Neocortical Epilepsy

**DOI:** 10.1038/s41598-019-41533-2

**Published:** 2019-03-27

**Authors:** Elvis Cela, Amanda R. McFarlan, Andrew J. Chung, Taiji Wang, Sabrina Chierzi, Keith K. Murai, P. Jesper Sjöström

**Affiliations:** 1 Brain Repair and Integrative Neuroscience Program, Centre for Research in Neuroscience, Departments of Medicine, and Neurology & Neurosurgery, The Research Institute of the McGill University Health Centre, Montreal General Hospital, Montreal, Quebec H3G 1A4 Canada; 2 Integrated Program in Neuroscience, McGill University, 3801 University Street, Montreal, Quebec H3A 2B4 Canada

## Abstract

Epileptogenesis is the gradual process by which the healthy brain develops epilepsy. However, the neuronal circuit changes that underlie epileptogenesis are not well understood. Unfortunately, current chemically or electrically induced epilepsy models suffer from lack of cell specificity, so it is seldom known which cells were activated during epileptogenesis. We therefore sought to develop an optogenetic variant of the classical kindling model of epilepsy in which activatable cells are both genetically defined and fluorescently tagged. We briefly optogenetically activated pyramidal cells (PCs) in awake behaving mice every two days and conducted a series of experiments to validate the effectiveness of the model. Although initially inert, brief optogenetic stimuli eventually elicited seizures that increased in number and severity with additional stimulation sessions. Seizures were associated with long-lasting plasticity, but not with tissue damage or astrocyte reactivity. Once optokindled, mice retained an elevated seizure susceptibility for several weeks in the absence of additional stimulation, indicating a form of long-term sensitization. We conclude that optokindling shares many features with classical kindling, with the added benefit that the role of specific neuronal populations in epileptogenesis can be studied. Links between long-term plasticity and epilepsy can thus be elucidated.

## Introduction

In 1967, Graham Goddard published his influential paper^1^ on the kindling model of epilepsy, where he described how brief daily high-frequency electrical stimulation of specific sub-cortical brain areas eventually led to behavioral seizures in a subset of otherwise healthy and non-epileptic animals. Because kindled animals retained a reduced threshold for seizures in the long term, Goddard argued that the process was “analogous to learning,”^1^ as proposed by Donald Hebb^2^ and others^3^, suggesting that epilepsy could arise from pathological activity patterns that recruit learning mechanisms in the healthy brain^4^. Although it does not represent all forms of epilepsy equally well^5^, the classical kindling model is today widely accepted as a functional epilepsy model in which pathological evoked activity gradually develops in otherwise healthy brains^6^.

Yet, the original kindling model suffers from a set of key problems. For example, it has been difficult to disentangle the contribution of tissue damage from plasticity mechanisms^7^. Furthermore, the experimenter cannot readily control the subset of cells that are activated with classical kindling, making it difficult to establish causal links between cell type and pathological outcome. This lack of specificity may in turn contribute to less standardized outcomes across labs.

To improve on these shortcomings, we developed an optogenetic kindling method. Optokindling shared several key features with the classical kindling model of epilepsy^1,4^: (1) repeated stimulation, while initially ineffective, eventually resulted in electrogaphic and behavioral seizures; (2) the severity and duration of these seizures increased over time; and finally, (3) animals with seizures that were left unstimulated for a prolonged period displayed retention of seizure potential when stimulation recommenced. Furthermore, optokindling was robust and did not cause appreciable brain damage or glial reactivity. Since the optically driven set of cells is genetically defined as well as fluorescently tagged in our model, it enables the study of cell and circuit changes associated with epileptogenesis.

## Results

### Establishing an optogenetic kindling approach

**Figure 1:**
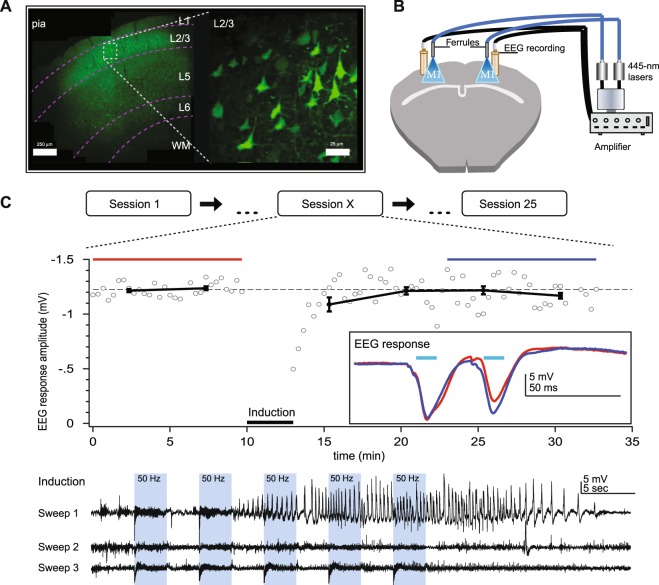
Optokindling via simultaneous EEG recording and ChR2 stimulation in awake behaving animals. (**A**) Coronal M1 section immunostained for EYFP indicated ChR2 expression in L2/3, 5, and 6, though predominantly in L2/3. Inset shows close-up of L2/3 ChR2-expressing PCs. (**B**) To simultaneously activate ChR2 and acquire EEG, ferrules and recording screws were implanted bilaterally above M1, without penetrating the cortex. Fiber optic cables were air-coupled to 445-nm lasers. EEG signals were processed by an extracellular amplifier, but not pre-amplified. A computer (not shown) TTL-gated the lasers and digitized amplified EEG signals. (**C**) In each stimulation session, M1 was kindled (during “Induction”) with 15 bouts of 3-second-long 50-Hz bursts of 5-ms 445-nm laser pulses, divided into three sweeps delivered once a minute. Sessions were repeated at least 25 times every two days. In this sample session from a non-naïve animal, a prominent electrographic seizure was evoked in the first induction sweep. EEG responses to 30-Hz paired-pulse laser stimuli were recorded for 10 minutes before and 20 minutes after the kindling induction. Data is represented as mean ± SEM here and throughout the manuscript unless stated otherwise. Inset: Paired-pulse EEG responses before (red) and after (blue) indicated a change in EEG dynamics but not amplitude.

To create an optogenetic variant of Goddard’s classical kindling model of epilepsy^1^, we expressed the high-efficiency E123T/T159C Channelrhodopsin-2 (ChR2) variant^8^ in M1 PCs using the CaMKIIα promoter, by bilateral stereotaxic injection of adeno-associated virus (AAV) in male P30–45 C57BL/6 mice (see Methods). We verified ChR2 expression by 2-photon laser-scanning microscopy (2PLSM) of the EYFP tag. This revealed dense expression in layer 2/3 (L2/3), sparse expression in L5 and L6, and no appreciable label in L1 or white matter (Fig. 1A), consistent with published expression patterns of the CaMKIIα promoter^9^.

We were concerned that the 445-nm laser light might not penetrate the entire cortical thickness, possibly leading to inefficient or unpredictable kindling. To investigate if light was delivered to subgranular layers in sufficient amounts to activate ChR2, we measured the transmission of light through neocortical tissue *ex vivo* (see Methods). We found that the penetration profile was consistent with previous reports^10^ (Supplementary Fig. S1). As a rule of thumb, light intensity must reach >1 mW/mm^2^ to ensure suprathreshold activation^11^. With dual 120-mW lasers and power loss through the fiber optic cable typically measured to <50% (data not shown), we used this profile (Supplementary Fig. S1) to estimate ~8 mW/mm^2^ at the L6/white-matter boundary, well above the intensity limit for suprathreshold activation. Furthermore, we note that the bulk of ChR2-expressing PCs were closer to the pial surface (Fig. 1), thus affording us a substantial safety margin.

We next wanted to verify that we could drive neocortical neurons at frequencies sufficient for kindling^12^. To do so, we used whole-cell recordings in acute slices (see Supplementary Methods) to explore the frequency dynamics of the high-efficiency E123T/T159C ChR2 variant^8^ that we used. We found that recorded L5 PCs followed 50-Hz light pulse trains with 90% fidelity (Supplementary Fig. S2), which should be more than adequate for binding neocortical PCs together by long-term potentiation (LTP)^13^ as well as for kindling^12^. Taken together (Supplementary Figs S1 and S2), our findings established that it should be possible to substitute optogenetic for electrical stimulation in the kindling model of epilepsy.

### Optogenetic and classical kindling share several hallmark features

We next kindled animals every two days with a brief laser stimulation paradigm (Fig. 1C). To quantify epileptogenesis, we recorded EEG with bilaterally implanted screw electrodes (Fig. 1B) and behavior with dual cameras, one placed above the animal (see Supplementary Movies 1 and 2), and one to the side. Inspired by classical *in-vitro* LTP experiments^13,14^, we also recorded EEG baseline responses before and after the kindling (Fig. 1C). This enabled us to look for long-term changes in amplitude and temporal dynamics of EEG responses (Fig. 1C; also see below and Fig. 5).

**Figure 2:**
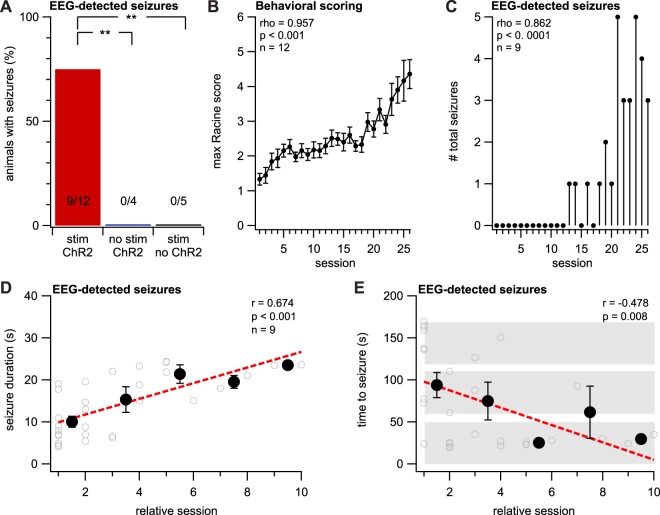
Optokindling and classical kindling share hallmark seizure features. (**A**) Optokindling required both laser stimulation and ChR2 expression. The number of stimulated ChR2-expressing animals that developed electrographic seizures (9 of 12 animals) was higher than unstimulated ChR2 controls (0 of 4) and stimulated no-ChR2 controls (0 of 5) (Fisher’s exact test, p = 0.001). (**B**) The behavioral seizure severity, as measured by a modified Racine score^17^, increased over sessions (Spearman’s rank correlation test, rho = 0.957, p < 0.001, n = 12 animals). (**C**) An increasing number of electrographic seizures developed in stimulated ChR2-expressing animals (Spearman’s rank correlation test, rho = 0.862, p < 0.001, n = 30 seizures from 9 animals). (**D**) Once seizures arose, seizure duration gradually increased over sessions (r = 0.674, p < 0.001, n = 30 seizures from 9 animals). Open circles represent individual seizures, whereas closed circles are averages over the one session before and after. Linear fits are made to the entire data set. (**E**) Seizure threshold, measured as time to electrographic seizure onset from start of induction, decreased across sessions (r = −0.478, p = 0.008, n = 30 seizures from 9 animals; symbols as in **D**). Gray boxes denote the three 50-Hz induction epochs. Linear fits were made to the individual data points, not binned data.

**Figure 3:**
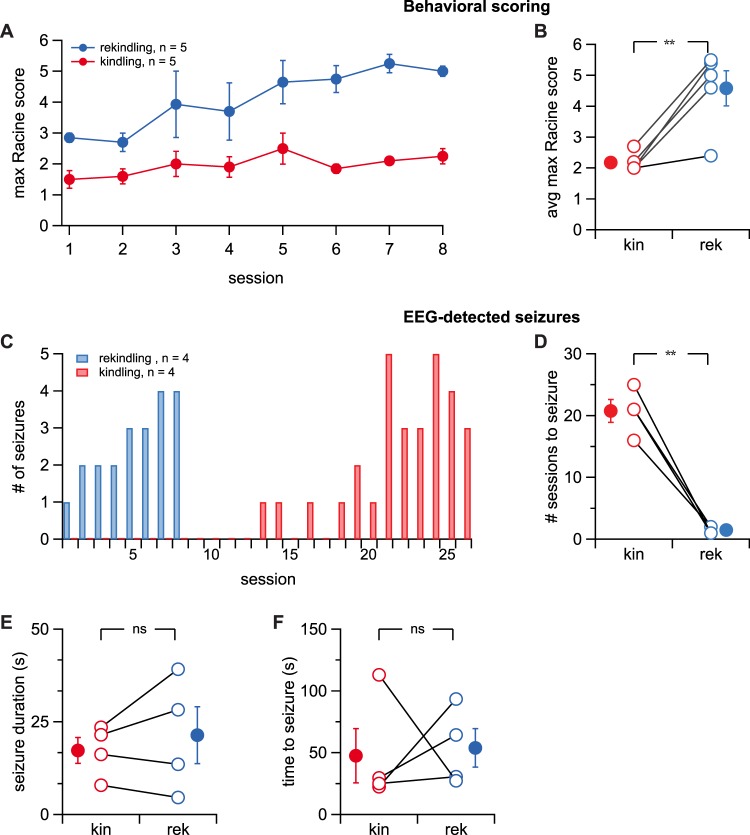
Kindled animals retained a long-term increase in seizure susceptibility. (**A**) Rekindled mice had more severe behavioral seizures compared to naïve animals (Kruskal-Wallis test, p < 0.001, n = 5 rekindled animals, n = 5 naïve animals). Racine scores from the eight rekindling sessions (blue) were compared with the first eight sessions in naïve animals (red). (**B**) Rekindled animals (“rek”) had more severe behavioral seizures than naïve animals (“kin”; Student’s paired *t* test, p = 0.009, n = 5 animals). (**C**) Electrographic seizures in rekindled animals occurred in earlier sessions than in naïve mice (Mann-Whitney’s U = 183.5, p < 0.001, n = 4 animals). (**D**) Electrographic seizures in rekindled mice occurred after fewer sessions than in naïve animals (Student’s paired *t* test, p = 0.003, n = 4 animals). (**E**) Electrographic seizure duration was indistinguishable between kindled and rekindled animals (Student’s paired *t* test, p = 0.43, n = 4 animals). (**F**) The seizure threshold, as measured by time to electrographic seizure onset after start of light stimulation (see Fig. 1C), was not different in kindled and rekindled mice (Student’s paired *t* test, p = 0.86, n = 4 animals).

**Figure 4:**
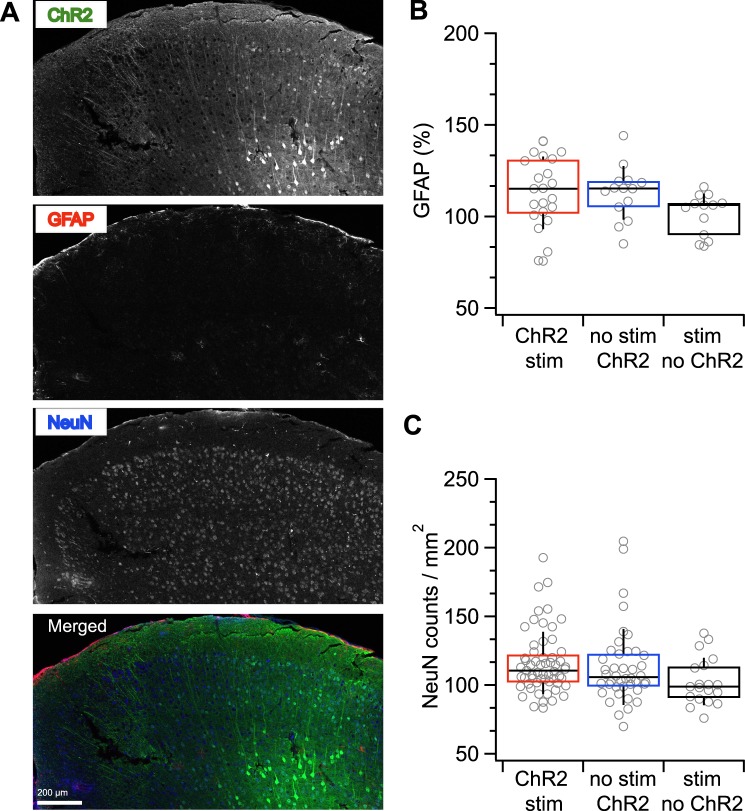
Immunohistology revealed no astrocytic reactivity or neuronal loss. (**A**) Sample coronal slices from an optogenetically kindled animal stained for EYFP to tag ChR2-expressing cells (“ChR2”), GFAP to label for astrocytic reactivity (“GFAP”), and NeuN to assess neuronal cell body counts (“NeuN”). (**B**) Astrocytic reactivity, as indicated by upregulated GFAP expression, was indistinguishable between animals with evoked seizures (“ChR2 stim”, n = 59 sections) and the two control groups (“no stim ChR2”, n = 42; “stim no ChR2”, n = 19; one-way ANOVA, p = 0.11). (**C**) Neuronal cell density did not differ between the three animal cohorts (“ChR2 stim”, n = 23, “no stim ChR2”, n = 14 and “stim no ChR2”, n = 13, one-way ANOVA, p = 0.10; compare Fig. 2A).

**Figure 5:**
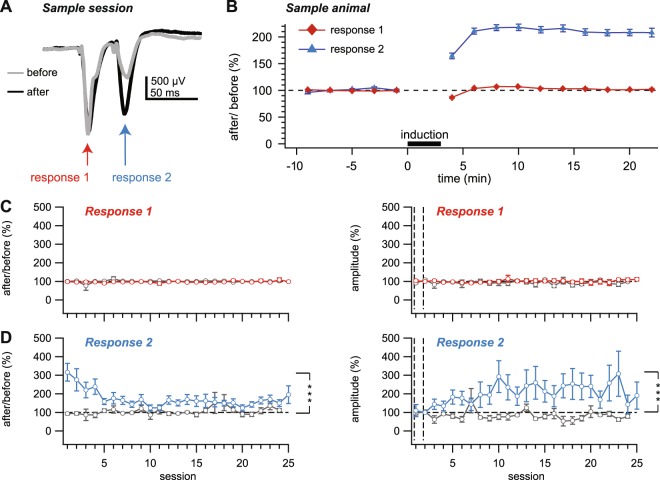
Evoked EEG responses exhibited long-term plasticity. (**A**) Example first and second EEG responses due to paired-pulse laser stimulation averaged during baseline periods before and after induction in one session. (**B**) Ensemble EEG response amplitude averaged across all sessions in one animal showed a within-session potentiation of the second but not the first response. (**C**) The magnitude of plasticity of the first EEG response remained unaffected across sessions and animals (left, p = 0.32, n = 9 stim ChR2 animals vs. n = 4 no stim ChR2 animals, Friedman test). The pre-induction baseline first response remained at the same amplitude across sessions and animals (right, responses normalized to the first two sessions indicated by vertical dashed lines, p = 0.99, stim ChR2 vs. no stim ChR2, Friedman test). Red: stim ChR2, gray: no stim ChR2. (**D**) The magnitude of plasticity of the second EEG response remained elevated across sessions and animals (left, p < 0.001, stim ChR2 vs. no stim ChR2, Friedman test), although waned in the first five sessions. The pre-induction baseline second response remained potentiated across sessions and animals (right, normalized as in C, p < 0.001, stim ChR2 vs. no stim ChR2, Friedman test), although seemed to saturate, perhaps as plasticity waned (left). Blue: stim ChR2, gray: no stim ChR2.

Evoked seizures were quite apparent, both behaviorally and electrographically (Supplementary Movie 2 and Fig. 1C). To eliminate experimenter bias associated with manual scoring, electrographic seizures were always automatically detected from EEG sweeps using a simple in-house software algorithm (Supplementary Fig. S3). Briefly, EEG spectral power was converted to z-score, and events exceeding a baseline-noise-determined z-score threshold for at least four seconds were automatically categorized as electrographic seizures (see Supplementary Methods). Properties such as seizure numbers and seizure duration were assessed using this automated analysis. Our custom software also enabled visual inspection by decomposing EEG signals into different frequency bands (Supplementary Fig. S3). Using our automated electrographic seizure detection in combination with our optokindling approach, we obtained evoked seizures in 9 out of 12 animals (Fig. 2A). As with classical electrical kindling^4^ (unless overkindled for long periods^15,16^), we never found spontaneous electrographic seizures, as based on a total of 125 hours of EEG recording.

To verify that the evoked seizures were specific for both laser stimulation and for ChR2 expression, we carried out two control experiments. First, we injected mice with ChR2-carrying AAV as before, but 50 Hz induction was omitted (Fig. 2A). Second, we injected mice with AAV carrying EYFP but no ChR2, and carried out induction as before (Fig. 2A). As expected, no electrographic seizures were detected in either control group (Fig. 2A). These control experiments furthermore verified the specificity of our automatic seizure detection algorithm (Supplementary Fig. S3).

We next explored if optokindling shared properties with classical kindling^1^. Using a modified version of the Racine scale^17^ (Table 1) to score behavior during the 3-minute-long induction period (see Methods and Fig. 1C), we found that severity increased over sessions (Fig. 2B**)**. In initial sessions, electrographic seizures were not detected, but they gradually emerged after several sessions (Fig. 2C**)**. With each session that passed, detected electrographic seizures increased in duration and were elicited after shorter periods of light stimulation (Fig. 2C,D). Since we stopped stimulation before Racine scores plateaued, it was not clear that behavioral seizure severity saturated in our experiments. Nevertheless, our optokindling approach thus shared several hallmark features with its classical counterpart^4^, including a gradual emergence in combination with increased seizure severity and duration, as well as lowered seizure threshold.

**Table 1:** The modified Racine scale used to score seizures.

Racine score	Description of behavior
1	Mouth and facial movements
2	Head nodding
3	Unilateral forelimb clonus
4	Bilateral forelimb clonus with rearing
5	Rearing and falling
6	Wild running, jumping, vocalizations

We used a modified variant of Racine’s scale^17,53^ to score evoked seizure behavior in parallel movies recorded with two cameras (see Methods).

It has been reported that seizure susceptibility has a circadian dependency^18^, preferentially striking during inactivity as related to the light-dark cycle. We found no correlation between the incidence of evoked seizures and circadian time, or between electrographic seizure duration and circadian time (Supplementary Fig. S4**)**. However, we did find a weak correlation between the time to electrographic seizure and circadian time (Supplementary Fig. S4), consistent with a lowered seizure threshold at night^18^.

### Optogenetically kindled mice retain a long-term susceptibility to seizures

A key feature of Goddard’s original model was that kindled animals retained a reduced threshold for seizures in the long term, which he interpreted as a form of memory^1^. We tested if this was true for our optokindling method as well. After successful kindling in a subset of animals, we halted stimulation for 36 days. We then reinitiated stimulation for a second rekindling period in the same animals. We pairwise compared rekindled animals with their naïve and kindled selves.

We found that seizures elicited in rekindled sessions were behaviorally more severe than in kindled sessions (Fig. 3A,B) and also occurred after fewer stimulation sessions (Fig. 3C,D). However, electrographic seizure duration and threshold did not change after rekindling compared to kindled mice (Fig. 3E,F). Because these longer-term experiments were prone to failure, the number of animals is lower, which means statistical power is relatively reduced. With that caveat, our results supported the view that optogenetically kindled animals retained a reduced threshold for seizures in the long term, as previously reported for classical kindling^1,4,6^.

### Seizures developed in the absence of gross brain damage and glial reactivity

Models of induced seizures can be associated with injury, which has been linked to higher seizure rates^19^. The contribution of pathological activity and plasticity to epileptogenesis can therefore be difficult to disentangle from that of injury. To assess the amount of tissue injury in our optokindling produced, we looked for reactive astrocytes and cell loss using GFAP and NeuN staining, respectively (see Supplementary Methods and Fig. 4). To visualize the region of ChR2-expressing cells, we stained for EYFP. We compared three categories of animals: optogenetically kindled animals, ChR2-expressing animals that were not kindled, and laser-stimulated animals that did not express ChR2 (the same three cohorts as in Fig. 2A). In comparing these three groups, we could not detect any differences in GFAP immunoreactivity or in neuronal cell counts (Fig. 4). In conclusion, pathological activity rather than injury, as indicated by astrocyte reactivity and neuronal cell loss, was the key causative agent in our optogenetic epilepsy model.

### Epileptogenesis is associated with long-term changes in EEG dynamics

Since Hebbian plasticity is intrinsically unstable^20^, we hypothesized that epileptogenesis might be driven by Hebbian plasticity^2^. If so, laser-light-evoked EEG responses should undergo long-lasting strengthening after optokindling^4,6^. To investigate if there were changes associated with the high-frequency induction stimulation, we compared EEG responses during the baseline periods after and before the induction (see Fig. 1C). To see if there were long-lasting changes that were maintained longer than the two-day spacing of sessions, we explored EEG responses during the pre-induction baseline period and compared them across all sessions.

We found that the first EEG response due to a paired-pulse laser stimulus was not potentiated (Fig. 5A–C), in disagreement with our hypothesis that classical Hebbian plasticity might underlie epileptogenesis in our optokindling model. The second EEG response, however, was strikingly potentiated within each session (Fig. 5B), as previously reported for hippocampal kindling^21^. The amount of potentiation of the second response gradually waned across sessions as the amplitude seemingly saturated (Fig. 5D). This potentiation was not due to developmental alteration in short-term dynamics previously found in juvenile necortex^22^, since it was absent in ChR2-expressing control animals that were not kindled (gray traces in Fig. 5).

Our results indicated that, although there was long-term plasticity of laser-evoked EEG responses, this probably did not correspond to Hebbian plasticity, since the second but not the first response due to a paired-pulse stimulus was potentiated. Perhaps long-term alterations of EEG dynamics were due to changes in synaptic short-term plasticity. Another not mutually exclusive possibility is a reduced inhibitory drive, as previously suggested^21^.

### Intrinsic cellular properties were not affected by kindling

Repeated high-frequency firing can alter the excitability of neocortical PCs^23^. We therefore wondered if optokindling gave rise to changes in intrinsic neuronal properties such as spike threshold, input resistance, or resting membrane potential. To explore this possibility, we recorded in acute slices the spiking responses of L2/3 PCs to gradually increasing current injections (Supplementary Fig. S5). We found, however, that none of the intrinsic cellular properties that we investigated were affected by the repeated high-frequency stimulation of optokindling (Supplementary Fig. S5).

### High-frequency power peaks before lower frequency power in evoked seizures

**Figure 6:**
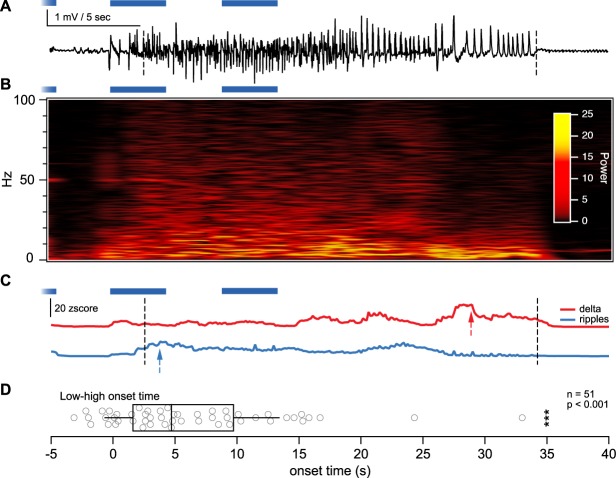
High-frequency oscillations peak before low-frequency oscillations. (**A**) Sample EEG trace illustrating an optogenetically evoked electrographic seizure, with automatically detected seizure start and end indicated by vertical dashed lines (see Methods and Supplementary Fig. S3). Note that seizure begins with rapid oscillations and ends with slow oscillations. Laser-light stimulation bouts are indicated in blue (Fig. 1C and Supplementary Fig. S3). (**B**) Wigner transform of electrographic seizure in (**A**) showing graded decay in high-frequency components as well as a gradual increase in low-frequency power. **(C)** FFT power z-score traces for delta (red, 4–8 Hz) and ripple bands (blue, 80–250 Hz) derived from the EEG trace in (**A**) shows how high-frequency oscillations peak (blue arrow) before their low-frequency counterparts (red arrow). (**D**) Peak power of high frequencies occurred earlier compared with that of low frequencies (6.4 ± 1 sec, n = 51 seizures from 9 animals, p < 0.001, *t* test for difference of mean compared to time zero). Data points indicate the difference between the low and high frequency peak times for individual seizures. Box plot shows first quartile, median, and third quartile with whiskers denoting one standard deviation from the mean.

It has previously been reported that high-frequency oscillations often precede the onset of neocortical electrographic seizures^24^. Visual inspection of seizures indicated that this could be the case in our model (Figs 1C and 6A–C; Supplementary Fig. S3A,C,D). If so, then the delta frequency peak in seizures should be preceded by a peak in the high-frequency ripple range (Fig. 6C). Indeed, we found that high-frequency power peaked more than six seconds before low-frequency power (Fig. 6D).

### Postictal depression of EEG power follows optogenetically induced seizures

Prior studies have revealed that EEG power is often temporarily reduced following electrographic seizures, a notion known as postictal depression^25^. In keeping with previous models of epilepsy^26^, we found a period of reduced EEG power following seizures. During this postictal period, EEG power was reduced by ~34% compared with preictal periods (see Supplementary Fig. S6).

### Evoked seizures do not affect EEG power in the long term

We next investigated how different frequency bands were individually altered by the kindling protocol over tens of minutes to days (Supplementary Fig. S7). We found that, after the induction (Fig. 1C), delta and theta band powers increased in power for many tens of minutes, while ripple and fast ripple band powers decreased (Supplementary Fig. S7). This effect did not, however, persist across stimulation sessions — no changes in the power of any frequency band was observed when comparing the baseline period before induction across sessions (Supplementary Fig. S7). This result was in contrast to that found with laser-light evoked EEG responses (Fig. 5), which were modified in the very long term, with changes persisting across sessions.

Taken together (Fig. 5, Supplementary Figs S6 and S7), our findings reveal a complex set of changes in intrinsic and evoked EEG dynamics, acting on different time scales. Although postictal depression of intrinsic EEG power followed immediately after optogenetically induced seizures (Supplementary Fig. S6), as previously shown^25,26^, this outcome depended on which frequency bands and on which time periods were analysed (Supplementary Fig. S7). Only the second of paired laser-evoked EEG responses were potentiated in the long term, persisting across different sessions (Fig. 5).

## Discussion

### Optokindling and classical electrical kindling share key properties

Based on the classical kindling model^1^, we developed a robust neocortical optogenetic kindling method of epilepsy. Optokindling recapitulated several essential hallmark features of its classical counterpart^4^. First, a majority of animals developed seizures, in line with electrical kindling studies showing robust seizure development^27,28^. Second, seizures emerged gradually over several stimulation sessions. Similar to electrical kindling, optokindling required more than ten sessions before first seizure and around twenty sessions for the development of generalized seizures^28,29^. Although some classical reports show generalized seizures earlier than session ten, there is considerable variability from animal to animal^30^. This is intriguing, given that electrical kindling stimulates a more diverse population of cells than our optokindling protocol. Third, once seizures began occurring, they increased in severity and duration across sessions. In addition, the threshold laser stimulation time necessary for evoking seizures decreased^4^, although we did not determine whether this also resulted in an extrafocal threshold reduction^31^. Finally, there was a long-term retention of seizure susceptibility in kindled animals. After a 36-day-long pause in stimulation, seizures were similar to those in kindled animals, suggesting that animals did not immediately ‘reset’ to the unkindled state once repeated stimulation sessions were halted. These findings are consistent with previous reports on the hallmark features of electrical kindling^27,32,33^.

As a proof of principle, we used our optogenetic model to demonstrate several findings previously found in epilepsy models. First, we found evidence that high-frequency oscillations precede low-frequency activity in seizures^24^. Second, we observed that postictal depression of EEG power was associated with seizures, as previously shown in neocortex^26^. Finally, we found that epileptogenesis was associated with a graded change in evoked EEG dynamics, as previously found in local field potentials of kindled hippocampus^21^.

### Advantages of optokindling

Optokindling has several advantages compared to its classical electric counterpart. First, seizures developed in the absence of gross brain damage, providing an experimental epileptogenesis paradigm with improved specificity for plasticity and for pathological activity. In the current study, viral injection was chosen as the method to restrict the population of cells expressing ChR2. This allowed us to more closely mimic the etiology of focal seizures which involves initial recruitment of a smaller network of neurons with subsequent spreading of activity and worsening of seizures. Although the initial viral injection presumably resulted in some degree of injury, we were not able to detect any many weeks afterwards. Importantly, injections of control AAV-EYFP did not result in any animals with seizures. In the future, ChR2-expressing transgenic mouse lines could be employed to eliminate any injury to the intact brain. Moreover, different number of cells could be progressively recruited during optokindling in ChR2-expressing transgenic mouse lines to investigate different epileptogenesis scenarios by dilating or restricting illumination area. Craniotomy can also be avoided completely by activating ChR2 through the skull^34,35^. Although several classical kindling studies also did not report gross tissue damage^19^, the chronic stimulation electrode must leave some damage whereas the fibers we used for optokindling did not penetrate the brain.

Second, optokindling enables targeted cell-type-specific recruitment. This is an important feature since neuronal plasticity depends on synapse type^36^, so kindling is expected to be cell-type dependent. Indeed, directly optogenetically driven seizures have been shown to depend critically on cell type^26^. Indiscriminate activation of several types of local neurons and fibers is in other words one drawback of classical electrical kindling, although optokindling will also drive other cell types indirectly. In the future, elucidating the cell-type dependence in optokindling will help clarify the circuit mechanisms that underpin epileptogenesis.

Third, optokindling provides a means for testing the two-hit model with respect to injury and inflammation. In the two-hit view on epilepsy, a second agent is required for spontaneous seizures to develop^37^. However, it is difficult to explore the involvement of inflammation and injury with classical electrical kindling, since the stimulation electrode introduces those two factors. With optokindling, in the absence of craniotomy or viral infection, inflammation could be systematically added as a second factor to investigate how it promotes epileptogenesis.

### Current limitations of optokindling

It is unclear why the expected spontaneous seizures seen in epilepsy are not observed with optokindling. However, it is noteworthy that spontaneous seizures have not been observed with classical electrical kindling either — unless the animal is over-kindled through hundreds of sessions^15,16^. This may imply that spontaneous seizures develop slowly. Alternatively, over-kindling with the classical protocol may contribute additional and possibly critically needed factors, e.g. injury or inflammation^19^ (although see ref.^38^). Optokindling may provide improved experimental control suitable for investigating the fundamental question of what is required to achieve spontaneous seizures.

Another limitation is that our optogenetic paradigm is time consuming. To enable us to monitor the gradual emergence of seizures, we spaced stimulation sessions by two days. We were also motivated by a concern that stimulation sessions spaced too closely might lower the efficacy of epileptogenesis^27,39^. Although this approach provided gradual emergence of evoked seizures, which was desirable for studying epileptogenesis, the long delay between sessions required more than 50 days of repeated spaced stimulation, which is not ideal for many applications. We note that this caveat is essentially shared with the classical electrical kindling model. We have, however, optimized optokindling using a stronger induction protocol to elicit seizures in <5 days (McFarlan *et al*., CAN 2018 poster 1-G-144). Additional work is required to narrow down the ideal parameter space for rapid optokindling.

### The role of plasticity in epileptogenesis

We demonstrated that due to repeated pathological activation of a small cluster of PCs in motor cortex, local circuits undergo plastic changes that lead to the appearance of generalized seizures. This circuit plasticity appeared to happen in the absence of gross brain damage and inflammation, implicating plasticity as key causative agent. Although all investigated EEG frequency bands were unaffected across sessions, we found long-term alterations of evoked EEG dynamics, additionally supporting the interpretation that plasticity at least partially underpinned the seizure-promoting circuit changes. However, since the initial amplitude of evoked EEG responses was unaffected, this did not appear to be a form of Hebbian plasticity^2^. Although we were inspired by classical LTP protocols in designing our optogenetic protocol, our study did not directly address how Hebbian LTP related to epileptogenesis^4,6^, which would have required testing if kindling occluded subsequent LTP induction^13,14^. We also explored whether optokindling was associated with changes in PC intrinsic properties, but found none, consistent with previous reports^40,41^. There were, however, marked changes in short-term dynamics of evoked EEG responses, which could have been due to alterations in synaptic short-term plasticity, or to reduced inhibitory feedback^21^. Plasticity was thus linked to epileptogenesis in our model, although the specific nature of this plasticity remains to be uncovered. This link is consistent with Goddard’s interpretation of the long-lasting increased susceptibility to seizures as a form of memory in the original kindling model^1^, since plasticity has been postulated to underlie learning^2,3^.

### Future directions

To our knowledge, our model is the first to systematically use optogenetics for neocortical kindling of awake behaving and otherwise healthy animals. One recent hippocampal study demonstrated the graded development of seizures using optogenetics^42^, but it did not explore if the elevated seizure susceptibility was retained in the long term like in the original kindling model^1^, nor was it possible to evaluate the behavioral component since the mice were sedated. There have also been several studies using optogenetics to halt^43–46^ and initiate seizures in hippocampus^47,48^ as well as cortex^26,49^. Although these studies demonstrated optogenetically elicited seizures, they did not show kindling, i.e. a demonstration of both gradual changes in seizure threshold and severity, which is essential for providing a link to plasticity. Also, these studies relied on optogenetic stimulation either in combination with classical induction models^45,50^ or with pre-existing disease phenotypes^49^, thus making it difficult to disentangle the role of plasticity from that of injury and inflammation in seizure development. However, with optokindling, it is possible to isolate the distinct role of plasticity in epileptogenesis.

To sum up, even though kindling does not model all variants of epilepsy equally well^5^, it is with optokindling possible to circumvent several limitations associated with other seizure induction models, e.g. unknown cell identity and cell cluster size. Future studies with optokindling may explore cell type specific contributions to epileptogenesis, or microcircuit plasticity associated with epileptogenesis, in addition to the roles of injury and inflammation in the two-hit model. Because of its more specific focus on plasticity, we believe optokindling will be useful for finding therapeutic treatments, to halt or slow down pathological plasticity in epileptogenesis.

## Methods

### Ethics

All procedures conformed to the standards and guidelines set in place by the *Canadian Council on Animal Care* (CCAC) and the *Montreal General Hospital Facility Animal Care Committee* (FACC), with the appropriate approved protocols for animal use. For surgeries, animals were anesthetized with isoflurane (CDMV Inc., St-Hyacinthe, QC, Canada). For collection of acute slices, mice were anesthetized with Avertin (Sigma Aldrich, Oakville, ON, Canada) or isoflurane and sacrificed once the hind-limb withdrawal reflex was lost. Every attempt was made to ensure minimum discomfort of the animals.

### Stereotaxic viral injections and EEG screw implantation

We targeted ChR2 to primary motor cortex (M1) of male C57BL/6 J mice aged postnatal day (P) 30–45 using bilateral stereotaxic injection of AAV-CaMKIIα-hChR2-E123T/T159C-p2A-EYFP (UNC Virus Core, North Carolina, USA), since two kindling sites are known to function better than one^51^. Males were chosen solely instead of females since the estrus cycle can affect seizure susceptibility^52^. Virus from plasmid constructs contributed by Karl Deisseroth was packaged by UNC Vector Core into serotype 5 AAVs. Using a stereotactic apparatus (Just for Mouse 5731, Stoelting Inc, Wood Dale, IL, USA), we injected 1.2 μL of virus bilaterally, following previously described procedures^34^. Injection coordinates relative to bregma were 1.1 mm anteroposterior, 1.5 mm mediolateral and 0.8 mm dorsoventral. We subsequently placed and cemented 1.25 mm ceramic ferrules (CFC-230, Thorlabs, Newton, NJ, USA) over the injected region of each hemisphere at the same coordinates as injection. Ferrules contained 200-μm-diameter 0.37-NA multimode fiber to allow light delivery. Coincident with viral injection and ferrule placement, we also implanted recording and ground screws (1/8″, 000–120 thread, 90910A600, McMaster-Carr, Elmhurst, Illinois, USA), which were stabilized with dental cement (Lang Dental, Wheeling, IL, USA). Animals were evaluated for correct placement with enhanced yellow fluorescent protein (EYFP) staining (see below). The coordinates for recording screws typically centered around ±3 mm mediolateral, −0.6 mm anteroposterior while the reference screws were placed at ±3 mm ML and −4 mm AP. The screws had conductive wire soldered on and were connected to gold-plated jacks (64–132, Warner Instruments, Hamden, CT, USA; or 33 × 1880, Newark Electronics, Pointe Claire, QC, Canada) for EEG recordings. We used a separate ground reference screw for the left and right hemisphere.

### *In-vivo* recordings and optogenetic stimulation

Animals were given 21 days to recover from surgery after which, they were habituated for three days to the recording setup before commencing stimulation sessions. The recording cage consisted of a 30 cm diameter wide and 40 cm tall Plexiglas cylinder covered with copper mesh and with a copper bottom. To reduce noise by serving as a Faraday cage, the copper plate and mesh of the cage were connected to the amplifier chassis ground. Two 445-nm blue lasers (Monopower-455-150-MM-TEC, Alphalas GmbH, Germany) on kinematic V-clamp post mounts (C1513/M, Thorlabs, Newton, NJ, USA) for ease of alignment, were air coupled (aspheric FC/PC fiber port PAF-X-18-PC-A with HCP mounting bracket, Thorlabs, Newton, NJ, USA) to two two-meter-long fiber-optic patch cords (M83L01, Thorlabs, Newton, NJ, USA), coupled to 1.25 mm bilaterally head-mounted ceramic ferrules (CFLC230-10, Thorlabs, Newton, NJ, USA). The implanted ferrule did not penetrate the brain. The light power exiting the fiber was $\gtrsim$5  mW; it was measured before and after every stimulation session to ensure it remained stable. EEGs were collected at 2–10 kHz with an extracellular amplifier (Model 1700, AM Systems, Sequim, WA, USA) and were digitized on a PCI-6221 board (National Instruments, Austin, TX, USA) using in-house software running in Igor Pro 6.37 (Wavemetrics Inc., Lake Oswego, OR, USA) on a desktop computer (Micro Tower Desktop PC SL-DK-H61MX-ID, SuperLogics, Natick, MA) running Windows XP SP7. This computer also episodically TTL-gated the two 445-nm lasers. Animal behavior was recorded with two cameras, one above the recording setup, and one to the side (Logitech C525 webcam, Tiger Direct.ca Inc., Calgary, AB, Canada). Video was acquired using iSpy software (Version 6.7.9, https://www.ispyconnect.com) running on the EEG acquisition computer. To score behavioral severity of seizures from these videos, we used a modified Racine scale **(**Table 1**)**. If behavior straddled two Racine stages, the score was taken as the intermediate value, e.g. 4.5 if between 4 and 5. As a form of verification, the Racine score was determined independently of the automated electrographic seizure detection. We did not categorize paw movement due to direct optogenetic activation as clonus, since it is a trivial and direct consequence of activation of motor programs that is unrelated to epilepsy. Animals without seizures were confirmed to have viral expression by staining for EYFP and correct placement was verified with EEG responses due to laser light stimulation.

Mice were stimulated every two days with the same protocol lasting ~33 minutes. Stimulation sessions were numbered sequentially starting at one. Each session consisted of an initial 10-minute-long baseline period, a three-minute-long induction, and a second 20-minute-long baseline. Baseline stimulation consisted of two 10-ms 445-nm laser pulses delivered at 30 Hz every 10 seconds. The induction period consisted of five bouts of three seconds of 50-Hz stimulation at 50% duty cycle and three second inter-bout interval, repeated three times once a minute (Fig. 1). We categorized frequency bands as follows (e.g. Supplementary Fig. S3): delta (0–4 Hz), theta (4–8 Hz), alpha (8–12 Hz), beta (12–30 Hz), gamma (30–80 Hz), ripples (80–250 Hz), and fast ripples (250–500 Hz).

We employed two types of control animals. In EYFP controls, EYFP alone was expressed using AAV5-CaMKIIα-EYFP (UNC Virus Core, North Carolina, USA), and the stimulation during baseline and induction was as described above. In ChR2 controls, the same ChR2 construct as stimulated animals was expressed, but the 50-Hz stimulation was omitted from the induction period; the baseline stimulation pattern was as above.

In rekindling experiments, mice were not stimulated for 36 days after the last of the initial 25 kindling sessions. Once stimulation was resumed, mice were stimulated every two days, as before. The first session after the 36-day-long pause was considered rekindling session one, with subsequent sessions numbered sequentially.

## Supplementary information

Supplementary information

Supplementary Movie 1. No seizures were elicited in session 3.

Supplementary Movie 2. Sample seizure evoked at session 15.
