# Supplementary material for: An Optogenetic Kindling Model of Neocortical Epilepsy
Source: Sci Rep. 2019 Mar 27;9:5236. doi: 10.1038/s41598-019-41533-2 (PMC6437216; doi:10.1038/s41598-019-41533-2)
Supplement: Supplementary file 1 — Supplementary information [file 41598_2019_41533_MOESM1_ESM.pdf]

SUPPLEMENTARY MATERIALS

An Optogenetic Kindling Model of Neocortical Epilepsy

Elvis Cela, Amanda McFarlan, Andrew J. Chung, Taiji Wang, Sabrina Chierzi,
Keith K. Murai, and P. Jesper Sjöström[1]

[1] Corresponding author

Email: jesper.sjostrom@mcgill.ca

Tel: +1-514-934-1934, x44561

Supplementary methods

Immunohistological analysis

Mice were anesthetized with a cocktail consisting of (in mg/mL) 50 Ketamine, 5 Xylazine, 1 Acepromazine (CDMV Inc., St-Hyacinthe, QC, Canada) and transcardially perfused with 0.1 M phosphate-buffered saline (PBS) followed by 4% paraformaldehyde (PFA, P6148, Sigma Aldrich, Oakville, ON, Canada). Brains were incubated in 4% PFA overnight, and then stored for two additional days in a 30% (w/v) sucrose solution. Next, brains were mounted in O.C.T. media (25608-930, VWR, Montreal, QC, Canada) using a bath of 100% EtOH and dry ice. Using a cryostat, embedded brains were sectioned at 50 µm thickness through the primary and secondary motor cortices and sections were placed in a 0.01 M PBS solution. Sections underwent a twenty-minute wash in 0.01 M PBS with 1% Triton-X (X100, Sigma Aldrich, Oakville, ON, Canada) followed by a ninety-minute wash in 0.01 M PBS with 0.3% Triton-X and 10% normal donkey serum (NDS) (566640, Sigma Aldrich, Oakville, ON, Canada). All antibody incubations were performed in 0.01 M PBS with 0.3% Triton-X and 1% NDS. Sections were incubated overnight at 4ºC in the primary antibodies: mouse anti-NeuN (1:500, Millipore, Billerica, MA, USA), rabbit anti-NeuN (1:500, Abcam, Cambridge, MA, USA), chicken anti-GFP (1:1000, Abcam, Cambridge, MA, USA), mouse anti-GFAP (1:500, Millipore, Billerica, MA, USA) and guinea pig anti-GFAP (1:500, Synaptic Systems, Göttingen, Germany). Twenty-four hours later, tissue underwent three 15-min washes in 0.01 M PBS with 0.3% Triton-X and 1% NDS, followed by a 90-min incubation in the Alexa Fluor donkey secondary antibodies at 1:1000 (anti-rabbit 405, anti-mouse 568, anti-rabbit 568, anti-chicken 488, anti-mouse 647, and anti-guinea pig 647) (Life Technologies, Burlington, ON, Canada). Next, the tissue underwent three twenty-minute washes in 0.01 M PBS with 0.3% Triton-X and 1% NDS.

Following this procedure, coronal slices were mounted using coverslips with a 40 µl bolus of ProLong Gold Antifade mount (P10144, Life Technologies, Burlington, ON, Canada). Sections were imaged using a Fluoview FV1000 confocal laser scanning microscope and Fluoview software (Olympus Canada, Richmond Hill, ON, Canada). Analysis of antibody-labelled slices was performed manually using ImageJ[54] and Igor Pro (Wavemetrics Inc., Lake Oswego, OR, USA). For GFAP, ~6 sections with two measurements each, covering all six cortical layers in M1, were analysed per animal. M1 was compared to a non-labelled cortical region. For NeuN, four or five sections were analysed with 7-8 measurements per animal. M1 cell counts were carried out across the six cortical layers.

Automated electrographic seizure detection

To independently detect seizures offline, a simple automatic seizure detection software algorithm was developed. Although this approach had the disadvantage of missing the occasional seizure that was detected by inspection, it was preferred because it was unbiased and — as opposed to manual inspection —invariably gave the same results when rerun on the same EEG sweeps. We manually inspected all automatically detected electrographic seizures.

EEG Fourier power traces were first converted to z-score sweeps. For z-scoring purposes, the background power levels were determined from the median of at least 64 one-second-long EEG segments recorded in the absence of laser stimulation. The median was used to automatically exclude the occasional movement artifact, because such artifacts resulted in massive responses that typically saturated the amplifier for about a second at a time.

To detect electrographic seizures, a combined threshold and duration criterion was applied. If power exceeded z-score 4 for longer than 4 seconds, then this event was deemed

a seizure. EYFP and ChR2 control animals were used to determine these threshold values, whereby which no seizures were detected in the control mice. We estimated a false negative rate of ~9% by direct inspection of EEGs. Z-scores above 100 were always rejected as movement artifacts. The z-score threshold crossing was taken as the start of an electrographic seizure. Seizure duration was automatically determined from seizure start to the first z-score downstroke threshold crossing. No attempt was made to merge events separated by a brief time of relative inactivity, so automatically detected electrographic seizures were likely underestimated both in terms of number and duration.

In-vitro acute slice experiments

Mice were anesthetized by an intraperitoneal injection of an Avertin solution (10 g 2,2,2-tribromoethyl alcohol, Sigma Aldrich T4,840-2, mixed with 10 ml of tert-amyl alcohol, Sigma Aldrich 24,048-6) at 500 mg/kg body weight. The animals were then perfused transcardially with an ice-cold (4˚C) oxygenated N-methyl-d-glucamine (NMDG) solution containing (in mM: 93 NMDG, 93 HCl, 2.5 KCl, 1.2 NaH_2PO_4, 30 $NaHCO_3$, 20 HEPES, 25 glucose, 5 sodium ascorbate, 3 sodium pyruvate, 10 $MgSO_4$ and 0.5 $CaCl_2$ bubbled with 95% O_2/5% CO_2[55]. After decapitation, the brain was removed and placed into the same cold NMDG solution. Coronal 300-µm-thick acute brain slices were prepared on a Campden Instruments 5000 mz-2 vibratome (Loughborough, UK) using ceramic blades (Lafayette Instrument, Lafayette, IN, USA). The brain slices were then kept at 33˚C in oxygenated NMDG solution for 10 minutes, after which they were transferred to an incubation solution (containing in mM: 125 NaCl, 2.5 KCl, 1 $MgCl_2$, 1.25 NaH_2PO_4, 2 $CaCl_2$, 26 $NaHCO_3$ and 25 glucose, bubbled with 95% O_2/5% CO_2) and kept for 1 hour before recording. Neurons were patched with pipettes (4-6 MΩ) filled with a gluconate-based current-clamp solution containing (in mM): 5 KCl, 115 K-gluconate, 10 K-HEPES,

4 Mg-ATP, 0.3 Na-GTP, 10 Na_2-phosphocreatine and 0.02-0.04 Alexa Fluor 594, adjusted to pH 7.2-7.4 with KOH and to 310 mOsm with sucrose. Patch pipettes were pulled from medium-wall capillaries using a P-1000 electrode puller (Sutter Instruments, Novato, CA, USA). Whole-cell recordings were obtained using BVC-700A (Dagan Corporation, Minneapolis, MN, USA). Voltage signals were digitized at 10 kHz using PCI-6229 boards (National Instruments, Austin, TX, USA) and custom software[13] running in Igor Pro (Wavemetrics Inc., Lake Oswego, OR, USA). L2/3 PCs were targeted for recording using a 2PLSM rig custom-built from a SliceScope (Scientifica, Uckfield, UK) as previously described[56]. A MaiTai HP laser (Spectraphysics, Santa Clara, CA, USA) tuned to 900-920 nm was used to excite GFP/Alexa 594 fluorophores.

To characterize ChR2 responses to light *in vitro* (**Supplementary Fig. S2**), we relied on the imaging and electrophysiology platform described above, with an additional 445-nm blue laser (eBay.ca, seller: Newgazer) guided onto the same light path as the 2-photon beam and controlled with two galvanometric mirrors (Cambridge Technologies, Bedford, MA, USA). The laser was controlled by the custom-made electrophysiology data acquisition software running in Igor Pro (see above).

Measurement of light scattering in cortical tissue

To measure light scattering properties in cortical tissue, we dissected whole brains from P30-45 mice and placed them on top of plain microscope slides (12-550-A3, Fisher Scientific, Nepean, ON, Canada). Next, we mounted the same 1.25-mm ferrule and the same 445-nm laser used for stimulation on the stereotax we performed our surgeries on. After, we mounted the sensor from a Thorlabs power meter (PM100D meter, S121C sensor, Thorlabs, Newton, NJ, USA) directly below the slide. We descended the ferrule into the brain in 100 μm increments while measuring the power that was able to reach the sensor.

We took 3 measurements at each depth and repeated the experiment with two different brains.

Statistics

The results are reported as the mean ± SEM. Significance levels are denoted using asterisks (* $p < 0.05$, ** $p < 0.01$, *** $p < 0.001$). Unless otherwise stated, we used Student's *t* test for equal means for all pairwise comparisons. If an equality of variances *F* test gave $p < 0.05$, we employed the unequal variances *t* test. Individual data sets were tested using a one-sample *t* test. For multiple comparisons, pairwise comparisons were carried out if one-way ANOVA suggested this at the $p < 0.05$ significance level. Equal or unequal variances ANOVA was used depending on Bartlett's test for equal variances. Wilcoxon-Mann-Whitney's non-parametric test was always used in parallel to the *t* test, with similar outcome. Multiple comparisons were corrected *post hoc* using Bonferroni-Dunn's method. Statistical tests were performed in Igor Pro 7 or 8 unless otherwise stated. For circular statistics (**Supplementary Fig. S4**), we used the *circstat* toolbox in MATLAB in conjunction with the Watson U^2 test[57].

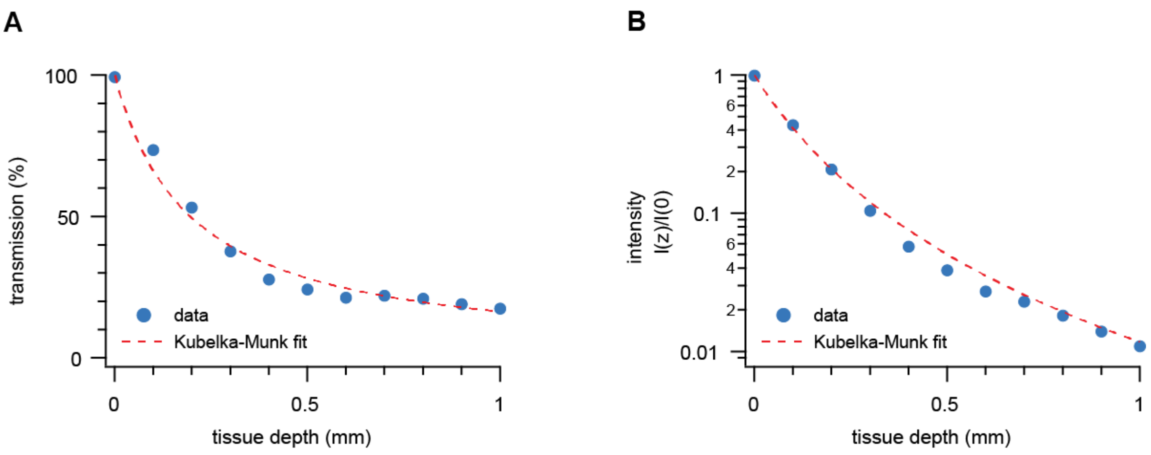

Supplementary figure 1. 445-nm laser light penetrated the neocortical thickness.

(A) The Kubelka-Munk model of light transmission through diffuse scattering media[58,59] fit our acquired transmission data well (scatter coefficient $S = 5.09$/mm, Chi^2 = 0.0013; n = 2 measurements per data point).

(B) Normalized light intensity as a function of the tissue depth, z, was also well fit by Kubelka-Munk's equation (scatter coefficient S = 4.35/mm, Chi^2 = 0.013). This treatment accounts for geometric loss and scattering of light for a 200-µm-diameter 0.37-NA multimode fiber[10].

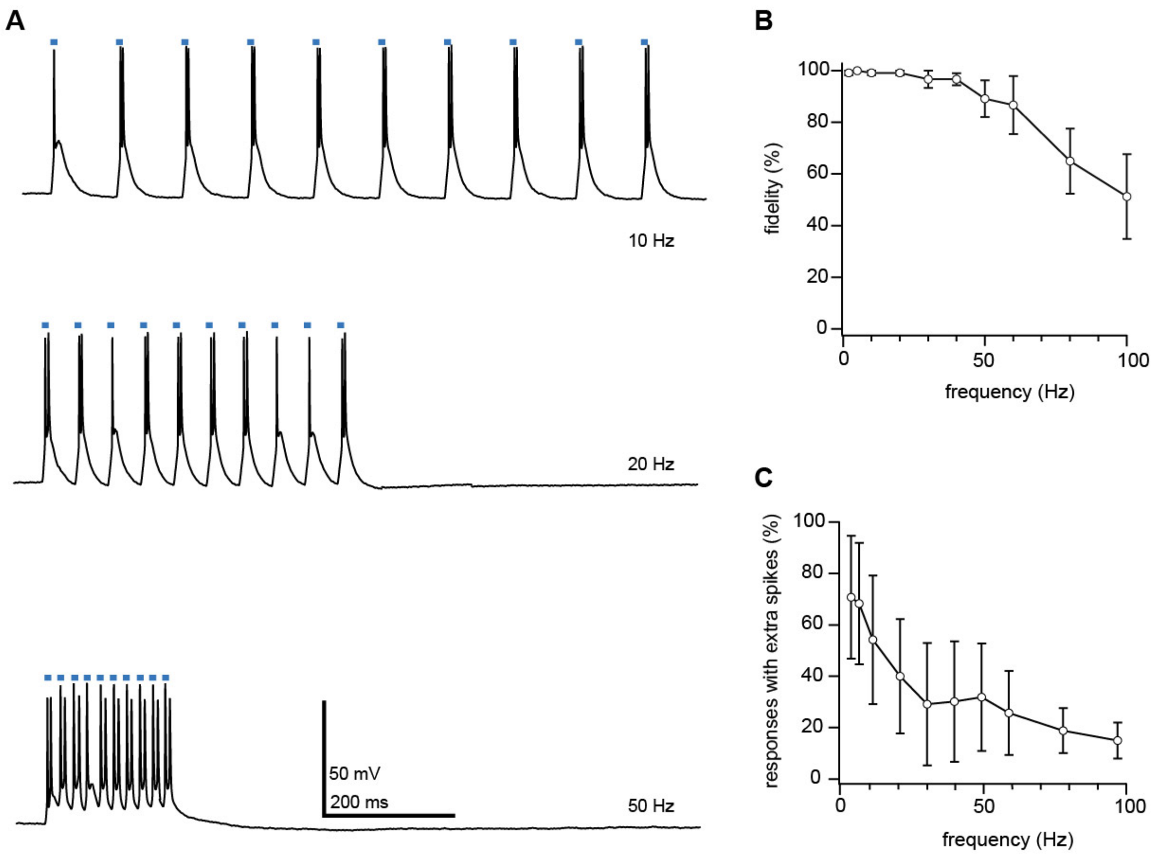

Supplementary figure 2. ChR2-expressing PCs can be reliably driven at 50 Hz.

(A) Representative spike trains in response to 50, 20 and 10 Hz light stimulation (blue lines) recorded from PCs in current clamp.

(B) Spiking was reliably evoked at stimulation frequencies up to 50 Hz (n = 4 cells, 89% ± 7% fidelity).

(C) Percent responses from (B) with extra spikes. Responses with one or more extra spikes per stimulus were counted once per response. Percentage was calculated as number of responses out of the total that had extra spikes in response to light stimulation per given frequency (n = 4 cells).

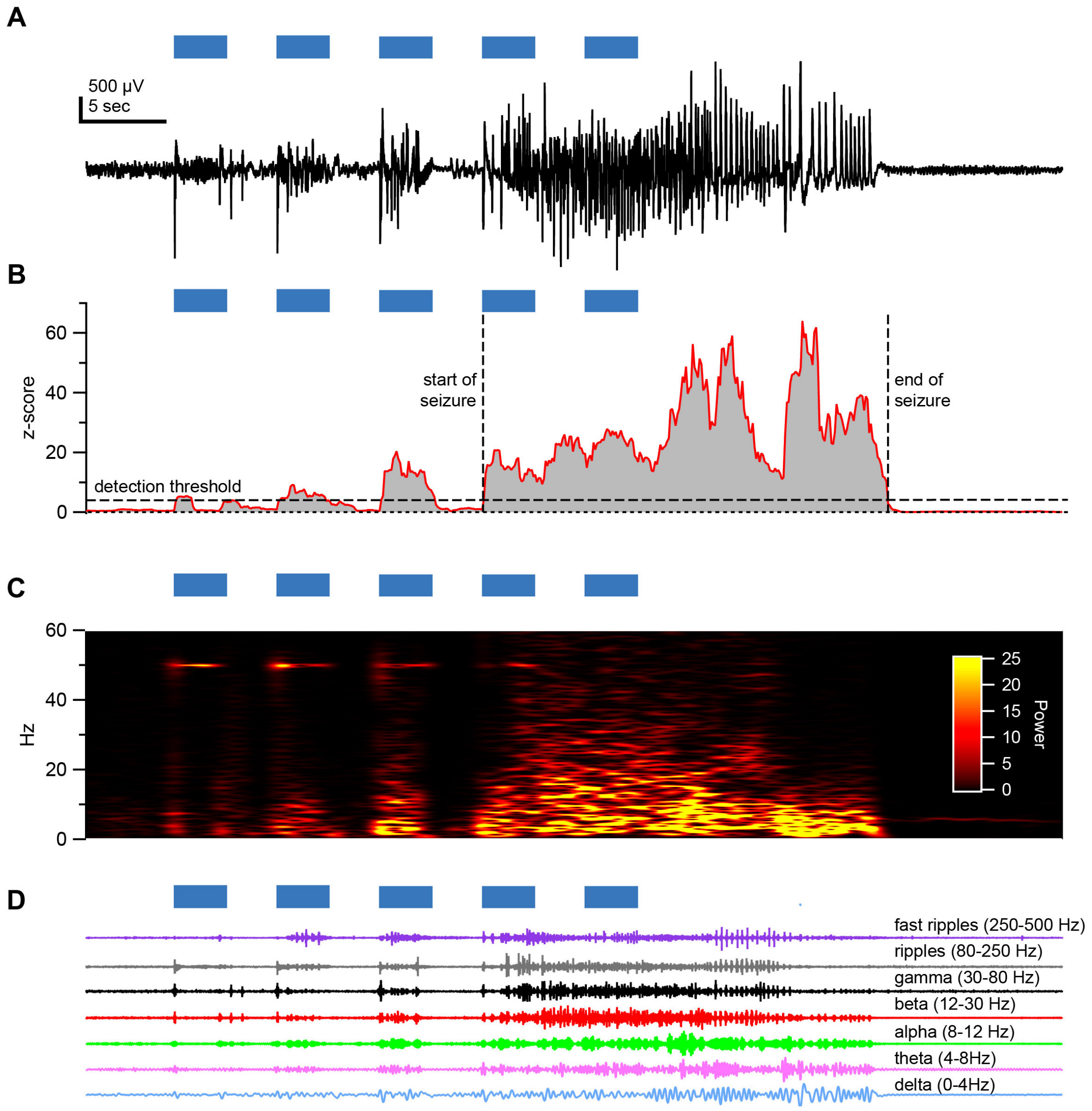

Supplementary figure 3. Automatic seizure detection.

(A) EEG of representative optogenetically evoked seizure with light stimulation depicted in blue. Seizure was evoked after several bouts of light stimulation.

(B) Corresponding z-score values from seizure in (A) showing duration of seizure between dashed lines (horizontal and vertical) used in detection algorithm for identifying seizures. Criteria for detection of the seizure in (A) was met both in duration and amplitude.

(C) Wigner transform of trace in (A) showing prominent power signature at 50 Hz as well as increases in lower power amplitudes as seizure emerges.

(D) Filtering of trace in (A) shows frequency bands from delta to fast ripples.

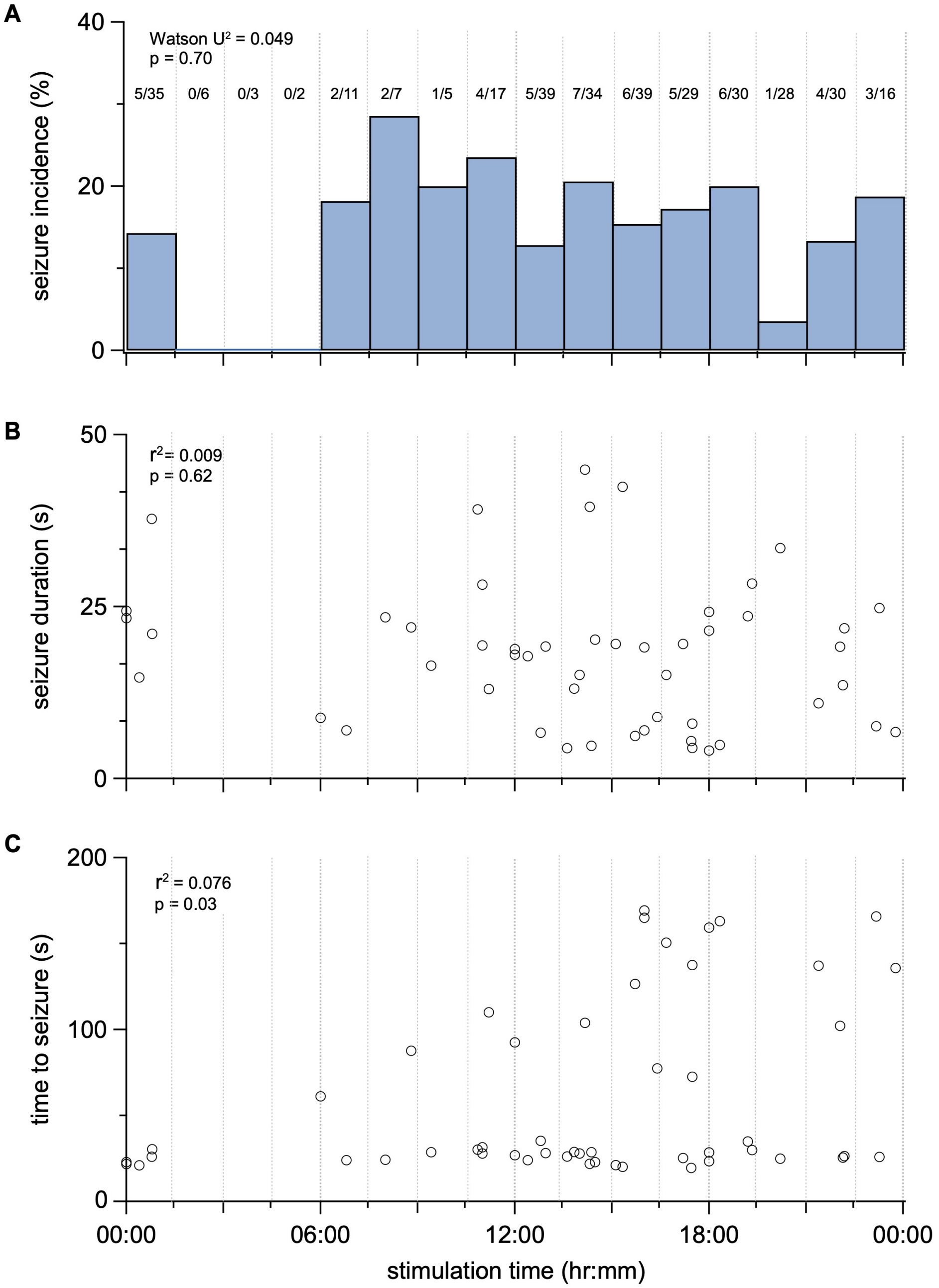

Supplementary figure 4. Seizure threshold depended weakly on circadian time.

(A) Incidence of evoked seizures was not correlated with circadian time (Watson's Two Sample U^2 test, p = 0.70, n = 51 seizures from 9 animals). Kindling and rekindling seizures were pooled.

(B) Seizure duration was not correlated with stimulation time (Circular-linear correlation, $r^2 = 0.009$, $p = 0.62$, $n = 51$ seizures from 9 animals).

(C) Time to seizure from light onset — a measure of seizure threshold (**Fig. 2)** — was weakly correlated with stimulation time (Circular-linear correlation, $r^{2\,=}\ 0.076$, $p = 0.03$, $n = 9$ animals).

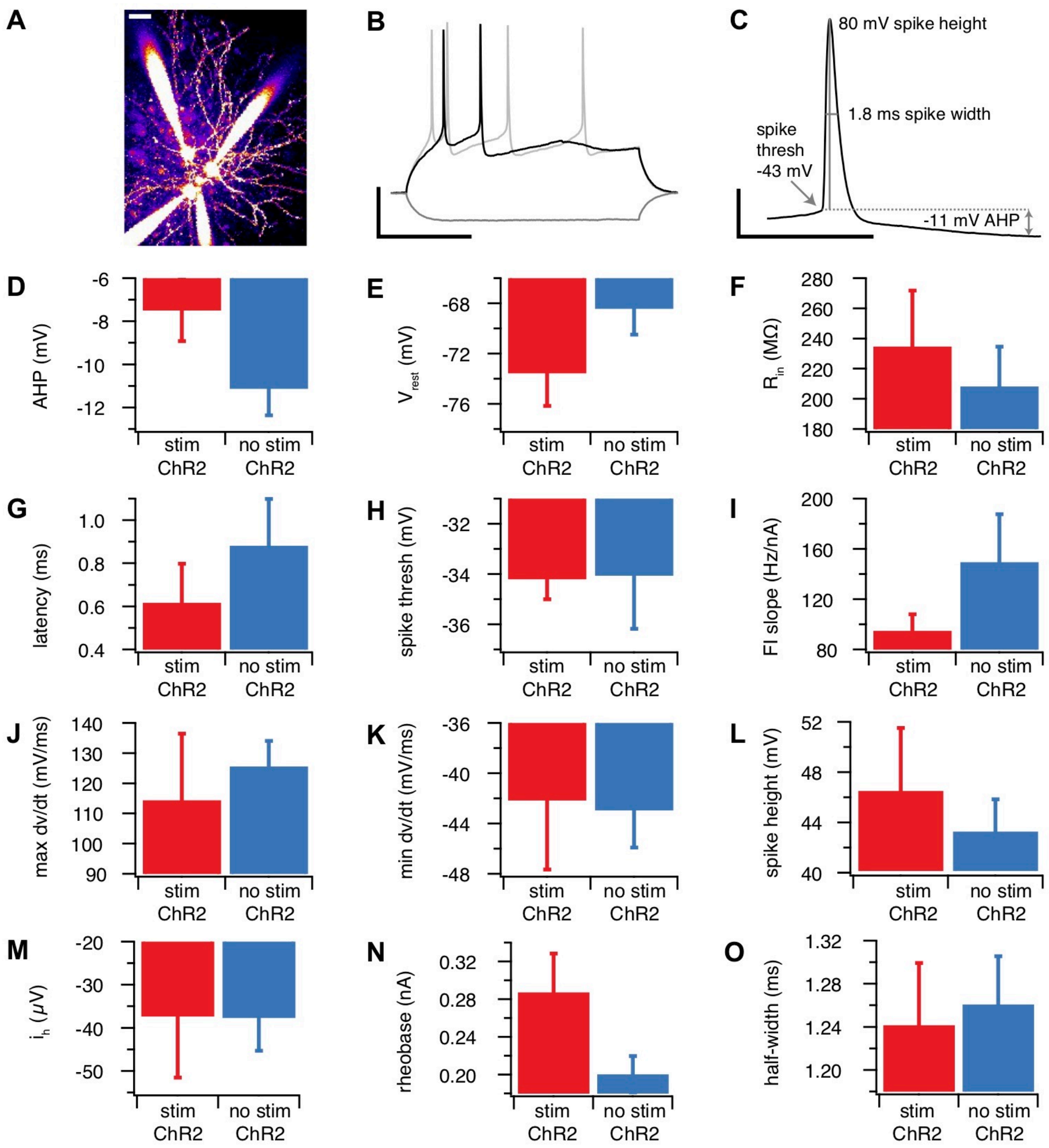

Supplementary figure 5. Intrinsic properties were indistinguishable in kindled and control animals.

(A) Sample flattened 2PLSM stack of Alexa-594-filled M1 L2/3 PCs. Scale bar: 20 μm.

(B) Three sample voltage traces recorded in an M1 L2/3 PC in response to a series of current pulses (rheobase sweep in black). The hyperpolarizing current was -100 pA, followed by a total of 14 depolarizing pulses starting at 100 pA, spaced by 20 pA up to

200 pA, and by 50 pA subsequently. From such sweeps, the intrinsic cellular properties were automatically extracted, as previously described[60]. Scale bar: 200 ms, 25 mV.

(C) Properties such as spike height, width, threshold (measured at inflexion point), and after-hyperpolarization (AHP, measured 30 ms after the inflexion point[61]) were automatically extracted[60] from the first action potential at rheobase (see panel B). Scale bar: 20 ms, 20 mV.

(D) AHPs in PCs recorded in acute slices from kindled ("stim ChR2", n = 8 cells, n = 3 animals) and control animals ("no stim ChR2", compare **Fig. 2A**, n = 17 cells, n = 3 animals) were indistinguishable (p = 0.067). No differences were found for any of the other intrinsic properties either: **(E)** resting membrane potential (V_{rest}, p = 0.133), **(F)** input resistance (R_{in}, p = 0.566), **(G)** latency to spike at rheobase (p = 0.359), **(H)** spike threshold (p = 0.947), **(I)** firing rate/current slope (p = 0.191), **(J)** maximum voltage time derivative (dv/dt, p = 0.637), **(K)** minimum voltage time derivative (p = 0.899), **(L)** spike height (p = 0.571), **(M)** hyperpolarization-activated current (I_h, measured as the sag at steady state due to hyperpolarizing step, p = 0.982), **(N)** rheobase current (p = 0.063), or **(O)** spike half-width (p = 0.793). Finally, series resistance (not shown, not compensated, measured from the fast component of a double-exponential fit to a hyperpolarizing pulse) was also indistinguishable (stim ChR2: 25 MΩ ± 3 MΩ vs. no stim ChR2: 23 MΩ ± 2 MΩ, p = 0.647). Student's *t* test was used throughout except panel N, where Wilcoxon's rank test was applied since current injections were distributed at discrete intervals.

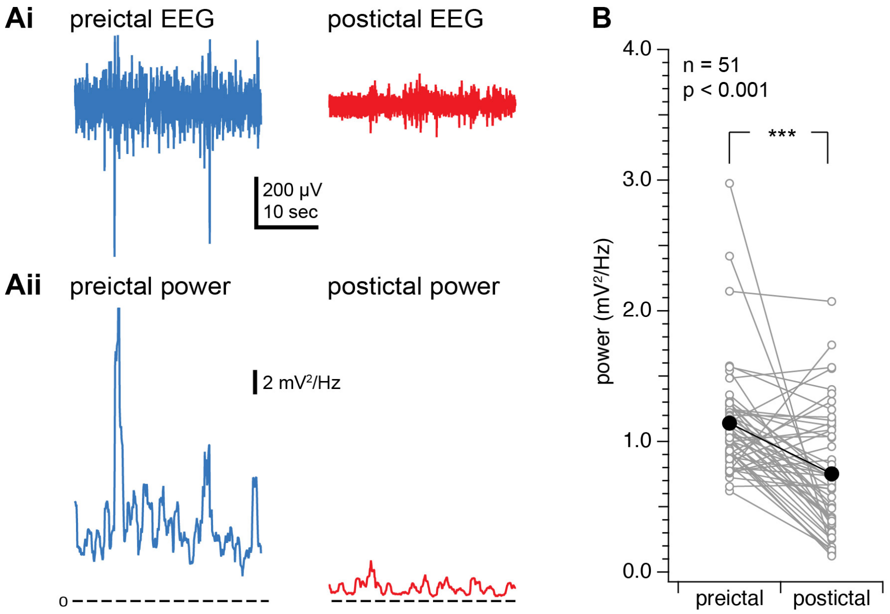

Supplementary figure 6. Post-ictal depression followed optogenetically elicited seizures.

(A) Sample preictal EEG trace (Ai) from a laser-stimulated ChR2-expressing animal was of considerably larger power (Aii) than the corresponding postictal EEG (Ai right, Aii right). Dashed lines in Aii indicate zero power level.

(B) Average EEG power was lower after (0.75 ± 0.06, n = 51 seizures from 9 animals) compared with before seizures (1.14 ± 0.06, Wilcoxon signed rank test, $p < 0.001$). EEG postictal and preictal power was measured 200 seconds after and before evoked seizures.

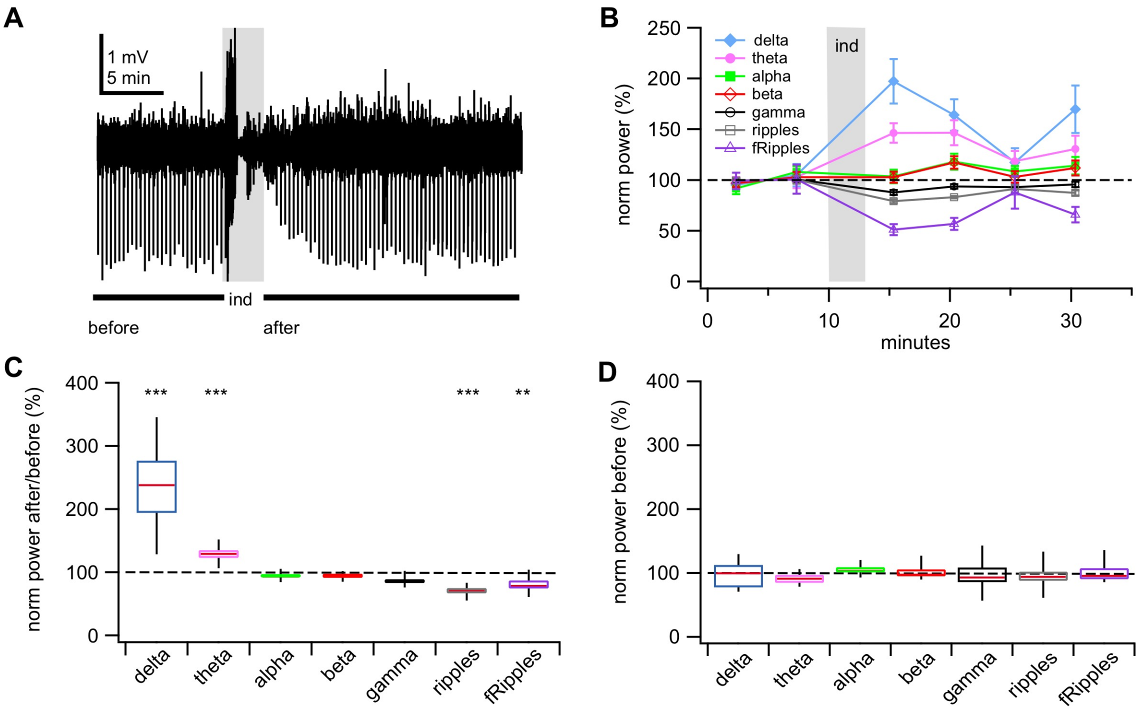

Supplementary figure 7. Frequency dynamics change within but not across sessions.

(**A**) Representative EEG traces showing periods before, during and after induction in an optogenetically-kindled, ChR2-expressing animal.

(**B**) Normalized power of different frequency bands over one stimulation session in one animal corresponding to the EEG shown in (A). Induction period where 50-Hz stimulation was applied is shaded in grey.

(**C**) To look for plasticity in the different frequency bands, we measured the power of each band over the stimulation period and compared the normalized power after induction with that before (as in **Supplementary Fig. S7B**), but across all sessions (similar to **Fig. 5C-D left**). Normalized power of delta and theta was elevated after induction while ripple and fast ripple power was reduced ($n = 9$ animals, 25 sessions/animal, one-way ANOVA, delta, theta, ripples $p < 0.001$, fast ripples $p = 0.007$, while alpha, beta, gamma $p > 0.05$), indicating a form of frequency band plasticity within sessions. Box plots show first

quartile, median, and third quartile with whiskers denoting one standard deviation from the mean.

(**D**) To see if the plasticity in frequency dynamics persisted across sessions, we measured the power of each frequency band in the baseline period before induction, averaged across sessions, and normalized to the power of each session to enable cross-animal comparison (analogous to **Fig. 5C-D right**). Normalized power did not change across sessions for any frequency band (n = 9 animals, one-way ANOVA, all bands $p > 0.05$), arguing against this possibility. Box plots are as in C.

Supplementary Movie 1. No seizures were elicited in session 3.

Sample video (top) and EEG recording (bottom, black trace) showing that in session 3, seizures were not evoked in this animal. The laser pulses are indicated in the bottom graph in blue, while the EEG total power is indicated in yellow. This session was scored as Racine stage 1 (**Table 1**).

Supplementary Movie 2. Sample seizure evoked at session 15.

Sample video (top) and EEG recording (bottom) of the same animal as in **Supplementary Movie 1** showing evoked seizure in session 15. This session was scored as Racine stage 5 (**Table 1**).

Supplementary References

54 Schneider, C. A., Rasband, W. S. & Eliceiri, K. W. NIH Image to ImageJ: 25 years of image analysis. *Nature methods* **9**, 671-675 (2012).

55 Ting, J. T., Daigle, T. L., Chen, Q. & Feng, G. Acute brain slice methods for adult and aging animals: application of targeted patch clamp analysis and optogenetics. *Methods Mol Biol* **1183**, 221-242, doi:10.1007/978-1-4939-1096-0_14 (2014).

56 Abrahamsson, T. *et al.* Differential Regulation of Evoked and Spontaneous Release by Presynaptic NMDA Receptors. *Neuron* **96**, 839-855 e835, doi:10.1016/j.neuron.2017.09.030 (2017).

57 Mardia, K. V. & Jupp, P. E. *Directional statistics*. (J. Wiley, 2000).

58 Kubelka, P. New contributions to the optics of intensely light-scattering materials. *J Opt Soc Am* **38**, 448-457 (1948).

59 Mobley, J. & Vo-Dinh, T. in *Biomedical Photonics Handbook* (CRC Press, 2003).

60 Buchanan, K. A. *et al.* Target-specific expression of presynaptic NMDA receptors in neocortical microcircuits. *Neuron* **75**, 451-466, doi:10.1016/j.neuron.2012.06.017 (2012).

61 Bean, B. P. The action potential in mammalian central neurons. *Nat Rev Neurosci* **8**, 451-465 (2007).
